# Poly[[tetra­aqua­bis­(μ_3_-imidazole-4,5-dicarboxyl­ato)tetra­kis­(μ_2_-imidazole-4,5-dicarboxyl­ato)tricobalt(II)dilutetium(III)] dihydrate]

**DOI:** 10.1107/S1600536811045764

**Published:** 2011-11-05

**Authors:** Li-Cai Zhu

**Affiliations:** a School of Chemistry and Environment, South China Normal University, Guangzhou 510631, People’s Republic of China

## Abstract

In the title compound, {[Co_3_Lu_2_(C_5_H_2_N_2_O_4_)_6_(H_2_O)_4_]·2H_2_O}_*n*_, the Lu^III^ ions are seven-coordinated in a monocapped trigonal prismatic coordination geometry by six O atoms from three imidazole-4,5-dicarboxyl­ate ligands and one water O atom. The Co^II^ ions are six-coordinated in a slightly distorted octa­hedral geometry and exhibit two types of coordination environments. One Co^II^ ion, located on an inversion center, is coordinated by two water O atoms as well as two O atoms and two N atoms from two imidazole-4,5-dicarboxyl­ate ligands. The other Co^II^ ion is bonded to four O atoms and two N atoms from four imidazole-4,5-dicarboxyl­ate ligands. These metal coordination units are connected by bridging imidazole-4,5-dicarboxyl­ate ligands, generating a three-dimensional network. The crystal structure is further stabilized by N—H⋯O, O—H⋯O, and C—H⋯O hydrogen-bonding inter­actions between the water mol­ecules and the imidazole-4,5-dicarboxyl­ate ligands.

## Related literature

For lanthanide–transition metal heterometallic complexes with bridging multifunctional organic ligands, see: Cheng *et al.* (2006 ▶); Kuang *et al.* (2007 ▶); Sun *et al.* (2006 ▶); Zhu *et al.* (2010 ▶).
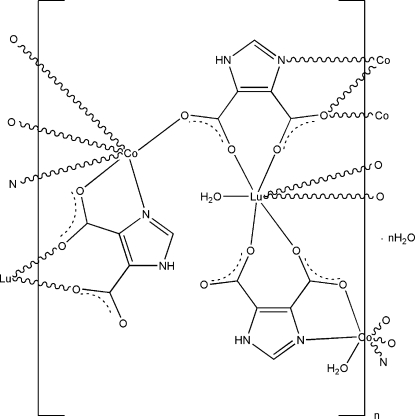


## Experimental

### 

#### Crystal data

[Co_3_Lu_2_(C_5_H_2_N_2_O_4_)_6_(H_2_O)_4_]·2H_2_O
*M* *_r_* = 1559.34
Triclinic, 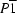

*a* = 7.0332 (6) Å
*b* = 8.3468 (7) Å
*c* = 17.8510 (15) Å
α = 95.515 (1)°
β = 96.786 (1)°
γ = 97.195 (1)°
*V* = 1025.84 (15) Å^3^
*Z* = 1
Mo *K*α radiation
μ = 6.08 mm^−1^
*T* = 296 K
0.20 × 0.18 × 0.15 mm

#### Data collection

Bruker APEXII area-detector diffractometer
Absorption correction: multi-scan (*SADABS*; Sheldrick, 1996 ▶) *T*_min_ = 0.308, *T*_max_ = 0.402
5351 measured reflections
3642 independent reflections
3280 reflections with *I* > 2σ(*I*)
*R*_int_ = 0.023

#### Refinement

*R*[*F* ^2^ > 2σ(*F* ^2^)] = 0.031
*wR*(*F* ^2^) = 0.069
*S* = 1.02
3642 reflections
376 parameters
12 restraints
H atoms treated by a mixture of independent and constrained refinement
Δρ_max_ = 1.38 e Å^−3^
Δρ_min_ = −1.34 e Å^−3^

### 

Data collection: *APEX2* (Bruker, 2004 ▶); cell refinement: *SAINT* (Bruker, 2004 ▶); data reduction: *SAINT*; program(s) used to solve structure: *SHELXS97* (Sheldrick, 2008 ▶); program(s) used to refine structure: *SHELXL97* (Sheldrick, 2008 ▶); molecular graphics: *XP* in *SHELXTL* (Sheldrick, 2008 ▶); software used to prepare material for publication: *SHELXL97*.

## Supplementary Material

Crystal structure: contains datablock(s) I, global. DOI: 10.1107/S1600536811045764/pv2457sup1.cif

Structure factors: contains datablock(s) I. DOI: 10.1107/S1600536811045764/pv2457Isup2.hkl

Additional supplementary materials:  crystallographic information; 3D view; checkCIF report

## Figures and Tables

**Table 1:** Hydrogen-bond geometry (Å, °)

*D*—H⋯*A*	*D*—H	H⋯*A*	*D*⋯*A*	*D*—H⋯*A*
N2—H1⋯O5^i^	0.86 (4)	2.04 (4)	2.900 (6)	175 (5)
N2—H1⋯O6^i^	0.86 (4)	2.59 (5)	3.139 (6)	122 (4)
O1*W*—H1*W*⋯O8^ii^	0.80 (5)	2.04 (5)	2.806 (6)	161 (6)
N4—H2⋯O1*W*^iii^	0.85 (4)	2.12 (3)	2.948 (6)	162 (5)
N4—H2⋯O10	0.85 (4)	2.57 (6)	3.016 (7)	114 (4)
O1*W*—H2*W*⋯O8^iv^	0.81 (5)	1.94 (6)	2.748 (6)	174 (6)
O2*W*—H3*W*⋯O3*W*	0.81 (5)	1.89 (5)	2.693 (6)	172 (7)
N5—H4⋯O8^v^	0.85 (4)	2.23 (4)	3.052 (6)	161 (5)
O2*W*—H4*W*⋯O10^vi^	0.80 (6)	2.06 (6)	2.851 (7)	171 (6)
O3*W*—H5*W*⋯O11^iii^	0.86 (5)	2.06 (6)	2.902 (6)	166 (5)
O3*W*—H6*W*⋯O4	0.86 (6)	2.10 (6)	2.928 (6)	163 (6)
C8—H8⋯O3	0.93	2.44	3.217 (7)	141
C8—H8⋯O9	0.93	2.39	3.190 (7)	144
C13—H13⋯O2*W*^vii^	0.93	2.42	3.352 (7)	178

Symmetry codes: (i) 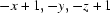; (ii) 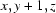; (iii) 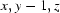; (iv) 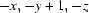; (v) 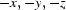; (vi) 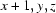; (vii) 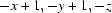.

**Fig. 1.**
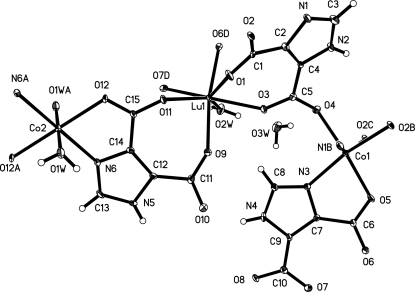
The asymmetric unit showing the atomic-numbering scheme and displacement ellipsoids drawn at the 30% probability level. Symmetry codes: A: 1 - x, 2 - y, -z; B: 1 - x, 1 - y, 1 - z; C: x, -1 + y, z; D: 1 + x, 1 + y, z.

**Fig. 2.**
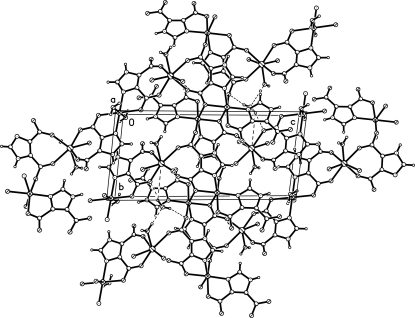
A view of the three-dimensional structure of the title compound, the hydrogen bonding interactions showed as broken lines.

## supplementary crystallographic information

### Comment

In the past few years, interest in the lanthanide-transition metal heterometallic complexes with bridging multifunctionnal organic ligands has been increasing, not only because of their impressive topological structures, but also due to their versatile applications in ion exchange, magnetism, bimetallic catalysis and luminescent probe (Cheng *et al.*, 2006; Kuang *et al.*, 2007; Sun *et al.*, 2006; Zhu *et al.*, 2010). As an extension of this research, the structure of the title compound, a new heterometallic coordination polymer, has been determined which is presented in this artcle.

The asymmetric unit of the title compound (Fig. 1), contains one Lu^III^ ions, two Co^II^ ions (one situated on an inversion centre), three imidazole-4,5-dicarboxylate ligands, two coordinated water molecules and one uncoordinated water molecule. The Lu^III^ ion is seven-coordinated in a monocapped trigonal prismatic coordination geometry by six O atoms from three imidazole-4,5-dicarboxylate ligands and one water O atom. Both Co^II^ ions are six-coordinated in a slightly distorted octahedral geometry. One Co^II^ ion lies on an inversion center and is coordinated with two O atoms from two coordinated water molecules as well as two O atoms and two N atoms from two imidazole-4,5-dicarboxylate ligands. The other Co^II^ ion is bonded to four O atoms and two N atoms from four imidazole-4,5-dicarboxylate ligands. These metal coordination units are connected by bridging imidazole-4,5-dicarboxylate ligands, generating a three-dimensional network (Fig. 2). The crystal structure is further stabilized by N—H···O, O—H···O, and C—H···O hydrogen-bonding interactions between water molecules, and imidazole-4,5-dicarboxylate ligands (Table 1).

### Experimental

A mixture of CoSO_4_.7H_2_O(0.141 g, 0.5 mmol), Lu_2_O_3_ (0.100 g, 0.25 mmol), imidazole-4,5-dicarboxylic acid (0.156 g, 1 mmol), and H_2_O (10 ml) was sealed in a 20 ml Teflon-lined reaction vessel at 443 K for 5 days then slowly cooled to room temperature. The product was collected by filtration, washed with water and air-dried. Red block crystals suitable for X-ray analysis were obtained.

### Refinement

H atoms bonded to C atoms were positioned geometrically and refined as riding, with C—H = 0.93 Å and *U*_iso_(H) = 1.2 *U*_eq_(C). H atoms bonded to N atoms and H atoms of water molecules were found from difference Fourier maps and refined isotropically with restraint:  N—H = 0.87 Å, O—H = 0.82 or 0.86 Å and *U*_iso_(H) = 1.5 *U*_eq_(N, O).

### Crystal data

**Table:**

[Co_3_Lu_2_(C_5_H_2_N_2_O_4_)_6_(H_2_O)_4_]·2H_2_O	*Z* = 1
*M**_r_* = 1559.34	*F*(000) = 751
Triclinic, *P*1	*D*_x_ = 2.524 Mg m^−^^3^
Hall symbol: -P 1	Mo *K*α radiation, λ = 0.71073 Å
*a* = 7.0332 (6) Å	Cell parameters from 2416 reflections
*b* = 8.3468 (7) Å	θ = 2.3–27.1°
*c* = 17.8510 (15) Å	µ = 6.08 mm^−^^1^
α = 95.515 (1)°	*T* = 296 K
β = 96.786 (1)°	Block, red
γ = 97.195 (1)°	0.20 × 0.18 × 0.15 mm
*V* = 1025.84 (15) Å^3^	

### Data collection

**Table:**

Bruker APEXII area-detector diffractometer	3642 independent reflections
Radiation source: fine-focus sealed tube	3280 reflections with *I* > 2σ(*I*)
graphite	*R*_int_ = 0.023
φ and ω scan	θ_max_ = 25.2°, θ_min_ = 2.3°
Absorption correction: multi-scan (*SADABS*; Sheldrick, 1996)	*h* = −5→8
*T*_min_ = 0.308, *T*_max_ = 0.402	*k* = −10→8
5351 measured reflections	*l* = −19→21

### Refinement

**Table:**

Refinement on *F*^2^	Primary atom site location: structure-invariant direct methods
Least-squares matrix: full	Secondary atom site location: difference Fourier map
*R*[*F*^2^ > 2σ(*F*^2^)] = 0.031	Hydrogen site location: inferred from neighbouring sites
*wR*(*F*^2^) = 0.069	H atoms treated by a mixture of independent and constrained refinement
*S* = 1.02	*w* = 1/[σ^2^(*F*_o_^2^) + (0.0315*P*)^2^ + 1.6377*P*] where *P* = (*F*_o_^2^ + 2*F*_c_^2^)/3
3642 reflections	(Δ/σ)_max_ = 0.001
376 parameters	Δρ_max_ = 1.38 e Å^−^^3^
12 restraints	Δρ_min_ = −1.34 e Å^−^^3^

### Special details

**Table:**

Geometry. All e.s.d.'s (except the e.s.d. in the dihedral angle between two l.s. planes) are estimated using the full covariance matrix. The cell e.s.d.'s are taken into account individually in the estimation of e.s.d.'s in distances, angles and torsion angles; correlations between e.s.d.'s in cell parameters are only used when they are defined by crystal symmetry. An approximate (isotropic) treatment of cell e.s.d.'s is used for estimating e.s.d.'s involving l.s. planes.
Refinement. Refinement of *F*^2^ against ALL reflections. The weighted *R*-factor *wR* and goodness of fit *S* are based on *F*^2^, conventional *R*-factors *R* are based on *F*, with *F* set to zero for negative *F*^2^. The threshold expression of *F*^2^ > σ(*F*^2^) is used only for calculating *R*-factors(gt) *etc*. and is not relevant to the choice of reflections for refinement. *R*-factors based on *F*^2^ are statistically about twice as large as those based on *F*, and *R*- factors based on ALL data will be even larger.

### Fractional atomic coordinates and isotropic or equivalent isotropic displacement parameters (Å^2^)

**Table:**

	*x*	*y*	*z*	*U*_iso_*/*U*_eq_	
Lu1	0.70112 (4)	0.58976 (3)	0.256536 (13)	0.01212 (9)	
Co1	0.38269 (11)	0.06475 (9)	0.42418 (4)	0.01307 (17)	
Co2	0.5000	1.0000	0.0000	0.0142 (2)	
C1	0.6258 (8)	0.7760 (7)	0.4223 (3)	0.0133 (12)	
C2	0.7053 (8)	0.6660 (7)	0.4742 (3)	0.0133 (12)	
C3	0.8108 (9)	0.6042 (7)	0.5848 (4)	0.0220 (14)	
H3	0.8561	0.6115	0.6362	0.026*	
C4	0.7216 (8)	0.5032 (6)	0.4657 (3)	0.0103 (11)	
C5	0.6582 (8)	0.3717 (7)	0.4028 (3)	0.0143 (12)	
C6	0.1022 (8)	−0.2023 (7)	0.3429 (3)	0.0132 (12)	
C7	0.1583 (8)	−0.1024 (6)	0.2827 (3)	0.0134 (12)	
C8	0.3107 (9)	0.1157 (7)	0.2497 (3)	0.0195 (13)	
H8	0.3898	0.2141	0.2508	0.023*	
C9	0.1057 (8)	−0.1066 (7)	0.2054 (3)	0.0138 (12)	
C10	−0.0137 (8)	−0.2248 (7)	0.1460 (3)	0.0149 (12)	
C11	0.2968 (9)	0.4493 (7)	0.1376 (3)	0.0172 (13)	
C12	0.3326 (8)	0.5562 (7)	0.0773 (3)	0.0138 (12)	
C13	0.2892 (9)	0.6358 (7)	−0.0373 (3)	0.0189 (13)	
H13	0.2474	0.6347	−0.0887	0.023*	
C14	0.4316 (8)	0.7088 (7)	0.0769 (3)	0.0135 (12)	
C15	0.5438 (8)	0.8341 (7)	0.1358 (3)	0.0140 (12)	
O1	0.5982 (6)	0.7412 (5)	0.3519 (2)	0.0215 (10)	
O2	0.5862 (6)	0.9071 (4)	0.4555 (2)	0.0147 (8)	
O3	0.6263 (6)	0.4072 (5)	0.3362 (2)	0.0195 (9)	
O4	0.6378 (6)	0.2288 (4)	0.4209 (2)	0.0192 (9)	
O5	0.1840 (6)	−0.1529 (5)	0.4094 (2)	0.0200 (9)	
O6	−0.0193 (6)	−0.3287 (5)	0.3281 (2)	0.0221 (10)	
O7	−0.1116 (6)	−0.3469 (5)	0.1661 (2)	0.0194 (9)	
O8	−0.0117 (6)	−0.2004 (5)	0.0777 (2)	0.0191 (9)	
O9	0.4324 (6)	0.4605 (5)	0.1932 (2)	0.0264 (10)	
O10	0.1470 (6)	0.3533 (5)	0.1285 (2)	0.0256 (10)	
O11	0.5857 (6)	0.8010 (5)	0.2037 (2)	0.0184 (9)	
O12	0.5894 (6)	0.9710 (5)	0.1153 (2)	0.0178 (9)	
N1	0.7588 (7)	0.7273 (6)	0.5495 (3)	0.0176 (11)	
N2	0.7899 (7)	0.4672 (6)	0.5366 (3)	0.0164 (11)	
N3	0.2873 (7)	0.0359 (5)	0.3091 (3)	0.0158 (11)	
N4	0.2033 (8)	0.0336 (6)	0.1876 (3)	0.0204 (11)	
N5	0.2432 (7)	0.5151 (6)	0.0041 (3)	0.0173 (11)	
N6	0.4021 (7)	0.7565 (5)	0.0052 (3)	0.0139 (10)	
H1	0.801 (9)	0.372 (4)	0.550 (3)	0.021*	
H2	0.209 (9)	0.060 (7)	0.1428 (17)	0.021*	
H4	0.181 (8)	0.420 (4)	−0.008 (3)	0.021*	
O1W	0.2273 (6)	1.0469 (5)	0.0245 (2)	0.0223 (10)	
H1W	0.160 (8)	0.965 (5)	0.030 (4)	0.033*	
H2W	0.168 (8)	1.090 (7)	−0.008 (3)	0.033*	
O2W	0.8496 (7)	0.3654 (5)	0.2236 (3)	0.0252 (10)	
H3W	0.826 (10)	0.284 (5)	0.244 (4)	0.038*	
H4W	0.931 (8)	0.351 (7)	0.197 (3)	0.038*	
O3W	0.8053 (7)	0.0904 (5)	0.2910 (3)	0.0314 (11)	
H5W	0.734 (8)	0.016 (7)	0.260 (3)	0.047*	
H6W	0.741 (9)	0.111 (8)	0.328 (3)	0.047*	

### Atomic displacement parameters (Å^2^)

**Table:**

	*U*^11^	*U*^22^	*U*^33^	*U*^12^	*U*^13^	*U*^23^
Lu1	0.01755 (14)	0.00981 (13)	0.00769 (13)	−0.00126 (9)	−0.00124 (9)	0.00199 (9)
Co1	0.0202 (4)	0.0086 (4)	0.0091 (4)	0.0011 (3)	−0.0020 (3)	0.0002 (3)
Co2	0.0174 (6)	0.0130 (5)	0.0121 (6)	0.0005 (4)	0.0001 (5)	0.0060 (4)
C1	0.016 (3)	0.014 (3)	0.010 (3)	−0.001 (2)	0.003 (2)	0.006 (2)
C2	0.015 (3)	0.015 (3)	0.011 (3)	0.005 (2)	0.006 (2)	−0.001 (2)
C3	0.030 (4)	0.018 (3)	0.017 (3)	0.007 (3)	−0.004 (3)	−0.001 (3)
C4	0.011 (3)	0.010 (3)	0.010 (3)	0.003 (2)	−0.002 (2)	0.004 (2)
C5	0.015 (3)	0.013 (3)	0.016 (3)	0.002 (2)	0.004 (2)	0.003 (2)
C6	0.015 (3)	0.014 (3)	0.011 (3)	0.003 (2)	0.002 (2)	0.003 (2)
C7	0.014 (3)	0.010 (3)	0.016 (3)	0.000 (2)	0.000 (2)	0.003 (2)
C8	0.029 (4)	0.013 (3)	0.014 (3)	−0.003 (3)	−0.004 (3)	0.004 (2)
C9	0.017 (3)	0.015 (3)	0.009 (3)	0.002 (2)	0.000 (2)	0.004 (2)
C10	0.013 (3)	0.018 (3)	0.015 (3)	0.006 (2)	0.001 (2)	0.003 (2)
C11	0.022 (3)	0.010 (3)	0.020 (3)	0.003 (2)	0.002 (3)	0.005 (2)
C12	0.012 (3)	0.015 (3)	0.013 (3)	0.001 (2)	−0.002 (2)	0.001 (2)
C13	0.022 (3)	0.021 (3)	0.012 (3)	0.003 (3)	−0.007 (3)	0.002 (2)
C14	0.011 (3)	0.015 (3)	0.014 (3)	0.003 (2)	−0.001 (2)	0.001 (2)
C15	0.019 (3)	0.013 (3)	0.009 (3)	0.000 (2)	0.000 (2)	0.002 (2)
O1	0.035 (3)	0.021 (2)	0.009 (2)	0.011 (2)	−0.0023 (18)	0.0024 (17)
O2	0.025 (2)	0.0101 (19)	0.0083 (19)	0.0051 (17)	−0.0017 (17)	−0.0002 (16)
O3	0.031 (2)	0.015 (2)	0.011 (2)	−0.0016 (18)	−0.0005 (18)	0.0032 (17)
O4	0.023 (2)	0.011 (2)	0.022 (2)	−0.0015 (17)	−0.0008 (19)	0.0044 (17)
O5	0.028 (2)	0.018 (2)	0.011 (2)	−0.0003 (18)	−0.0064 (18)	0.0051 (17)
O6	0.027 (2)	0.018 (2)	0.017 (2)	−0.0079 (19)	−0.0043 (19)	0.0075 (18)
O7	0.025 (2)	0.015 (2)	0.015 (2)	−0.0040 (18)	0.0003 (18)	−0.0023 (17)
O8	0.024 (2)	0.022 (2)	0.009 (2)	−0.0009 (18)	−0.0021 (18)	0.0035 (17)
O9	0.031 (3)	0.020 (2)	0.023 (2)	−0.0083 (19)	−0.012 (2)	0.0112 (19)
O10	0.021 (2)	0.027 (2)	0.026 (2)	−0.006 (2)	−0.003 (2)	0.011 (2)
O11	0.033 (2)	0.013 (2)	0.009 (2)	0.0025 (18)	−0.0006 (18)	0.0029 (16)
O12	0.025 (2)	0.013 (2)	0.015 (2)	−0.0015 (17)	−0.0017 (18)	0.0090 (17)
N1	0.025 (3)	0.017 (3)	0.011 (2)	0.003 (2)	−0.001 (2)	0.002 (2)
N2	0.024 (3)	0.012 (2)	0.014 (3)	0.008 (2)	−0.002 (2)	0.007 (2)
N3	0.021 (3)	0.012 (2)	0.012 (2)	−0.003 (2)	−0.001 (2)	0.000 (2)
N4	0.026 (3)	0.024 (3)	0.013 (3)	0.001 (2)	0.005 (2)	0.011 (2)
N5	0.022 (3)	0.013 (2)	0.012 (3)	−0.004 (2)	−0.005 (2)	−0.004 (2)
N6	0.017 (3)	0.016 (3)	0.008 (2)	0.001 (2)	0.002 (2)	0.003 (2)
O1W	0.020 (2)	0.027 (3)	0.023 (2)	0.0045 (19)	0.0031 (19)	0.014 (2)
O2W	0.035 (3)	0.018 (2)	0.026 (3)	0.007 (2)	0.013 (2)	0.006 (2)
O3W	0.042 (3)	0.020 (2)	0.032 (3)	0.001 (2)	0.014 (2)	−0.001 (2)

### Geometric parameters (Å, °)

**Table:**

Lu1—O9	2.183 (4)	C7—N3	1.382 (7)
Lu1—O6^i^	2.206 (4)	C8—N3	1.320 (7)
Lu1—O3	2.238 (4)	C8—N4	1.340 (8)
Lu1—O7^i^	2.261 (4)	C8—H8	0.9300
Lu1—O1	2.264 (4)	C9—N4	1.366 (7)
Lu1—O11	2.270 (4)	C9—C10	1.477 (8)
Lu1—O2W	2.317 (4)	C10—O8	1.257 (7)
Co1—N3	2.066 (5)	C10—O7	1.263 (7)
Co1—O2^ii^	2.121 (4)	C11—O10	1.226 (7)
Co1—O5	2.123 (4)	C11—O9	1.281 (7)
Co1—O2^iii^	2.125 (4)	C11—C12	1.486 (8)
Co1—O4	2.126 (4)	C12—C14	1.374 (8)
Co1—N1^ii^	2.147 (5)	C12—N5	1.374 (7)
Co2—N6	2.077 (4)	C13—N6	1.316 (7)
Co2—N6^iv^	2.077 (4)	C13—N5	1.335 (8)
Co2—O1W^iv^	2.092 (4)	C13—H13	0.9300
Co2—O1W	2.092 (4)	C14—N6	1.377 (7)
Co2—O12^iv^	2.126 (4)	C14—C15	1.486 (7)
Co2—O12	2.126 (4)	C15—O12	1.249 (6)
C1—O1	1.249 (6)	C15—O11	1.278 (6)
C1—O2	1.272 (6)	O2—Co1^ii^	2.121 (4)
C1—C2	1.478 (8)	O2—Co1^v^	2.125 (4)
C2—C4	1.374 (7)	O6—Lu1^vi^	2.206 (4)
C2—N1	1.383 (7)	O7—Lu1^vi^	2.261 (4)
C3—N1	1.323 (8)	N1—Co1^ii^	2.147 (5)
C3—N2	1.344 (7)	N2—H1	0.87 (2)
C3—H3	0.9300	N4—H2	0.85 (2)
C4—N2	1.374 (7)	N5—H4	0.85 (2)
C4—C5	1.479 (7)	O1W—H1W	0.80 (5)
C5—O3	1.256 (7)	O1W—H2W	0.81 (5)
C5—O4	1.260 (7)	O2W—H3W	0.81 (5)
C6—O6	1.258 (7)	O2W—H4W	0.80 (6)
C6—O5	1.262 (6)	O3W—H5W	0.86 (5)
C6—C7	1.481 (7)	O3W—H6W	0.86 (6)
C7—C9	1.382 (8)		
			
O9—Lu1—O6^i^	168.24 (15)	O6—C6—C7	121.6 (5)
O9—Lu1—O3	80.23 (15)	O5—C6—C7	116.0 (5)
O6^i^—Lu1—O3	89.91 (15)	C9—C7—N3	109.7 (5)
O9—Lu1—O7^i^	104.39 (15)	C9—C7—C6	136.0 (5)
O6^i^—Lu1—O7^i^	80.05 (14)	N3—C7—C6	114.2 (5)
O3—Lu1—O7^i^	144.82 (15)	N3—C8—N4	109.9 (5)
O9—Lu1—O1	103.10 (16)	N3—C8—H8	125.1
O6^i^—Lu1—O1	80.68 (15)	N4—C8—H8	125.1
O3—Lu1—O1	77.20 (14)	N4—C9—C7	103.9 (5)
O7^i^—Lu1—O1	132.94 (14)	N4—C9—C10	121.1 (5)
O9—Lu1—O11	80.95 (15)	C7—C9—C10	134.9 (5)
O6^i^—Lu1—O11	110.81 (14)	O8—C10—O7	122.9 (5)
O3—Lu1—O11	140.82 (15)	O8—C10—C9	118.6 (5)
O7^i^—Lu1—O11	73.48 (15)	O7—C10—C9	118.5 (5)
O1—Lu1—O11	74.08 (14)	O10—C11—O9	125.5 (5)
O9—Lu1—O2W	88.27 (17)	O10—C11—C12	118.2 (5)
O6^i^—Lu1—O2W	82.72 (17)	O9—C11—C12	116.1 (5)
O3—Lu1—O2W	73.16 (15)	C14—C12—N5	104.4 (5)
O7^i^—Lu1—O2W	72.16 (15)	C14—C12—C11	134.2 (5)
O1—Lu1—O2W	145.87 (15)	N5—C12—C11	121.2 (5)
O11—Lu1—O2W	139.98 (15)	N6—C13—N5	110.3 (5)
N3—Co1—O2^ii^	167.15 (17)	N6—C13—H13	124.8
N3—Co1—O5	76.85 (16)	N5—C13—H13	124.8
O2^ii^—Co1—O5	95.95 (15)	C12—C14—N6	109.5 (5)
N3—Co1—O2^iii^	112.98 (17)	C12—C14—C15	135.0 (5)
O2^ii^—Co1—O2^iii^	76.14 (16)	N6—C14—C15	115.2 (5)
O5—Co1—O2^iii^	83.14 (15)	O12—C15—O11	123.1 (5)
N3—Co1—O4	97.10 (17)	O12—C15—C14	116.4 (5)
O2^ii^—Co1—O4	93.03 (15)	O11—C15—C14	120.5 (5)
O5—Co1—O4	160.66 (16)	C1—O1—Lu1	142.1 (4)
O2^iii^—Co1—O4	82.44 (15)	C1—O2—Co1^ii^	117.9 (3)
N3—Co1—N1^ii^	95.80 (18)	C1—O2—Co1^v^	132.2 (4)
O2^ii^—Co1—N1^ii^	76.66 (16)	Co1^ii^—O2—Co1^v^	103.86 (16)
O5—Co1—N1^ii^	111.04 (17)	C5—O3—Lu1	144.9 (4)
O2^iii^—Co1—N1^ii^	150.45 (16)	C5—O4—Co1	130.4 (4)
O4—Co1—N1^ii^	87.67 (17)	C6—O5—Co1	117.4 (4)
N6—Co2—N6^iv^	180.0 (2)	C6—O6—Lu1^vi^	138.2 (4)
N6—Co2—O1W^iv^	93.28 (18)	C10—O7—Lu1^vi^	139.6 (4)
N6^iv^—Co2—O1W^iv^	86.72 (18)	C11—O9—Lu1	149.6 (4)
N6—Co2—O1W	86.72 (18)	C15—O11—Lu1	134.8 (4)
N6^iv^—Co2—O1W	93.28 (18)	C15—O12—Co2	116.7 (4)
O1W^iv^—Co2—O1W	180.0	C3—N1—C2	105.8 (5)
N6—Co2—O12^iv^	102.48 (16)	C3—N1—Co1^ii^	136.7 (4)
N6^iv^—Co2—O12^iv^	77.52 (16)	C2—N1—Co1^ii^	110.0 (4)
O1W^iv^—Co2—O12^iv^	91.60 (16)	C3—N2—C4	107.8 (5)
O1W—Co2—O12^iv^	88.40 (16)	C3—N2—H1	124 (4)
N6—Co2—O12	77.52 (16)	C4—N2—H1	127 (4)
N6^iv^—Co2—O12	102.48 (16)	C8—N3—C7	106.3 (5)
O1W^iv^—Co2—O12	88.40 (16)	C8—N3—Co1	138.1 (4)
O1W—Co2—O12	91.60 (16)	C7—N3—Co1	115.6 (4)
O12^iv^—Co2—O12	180.0 (2)	C8—N4—C9	110.2 (5)
O1—C1—O2	123.3 (5)	C8—N4—H2	124 (4)
O1—C1—C2	122.4 (5)	C9—N4—H2	126 (4)
O2—C1—C2	114.3 (5)	C13—N5—C12	109.3 (5)
C4—C2—N1	109.3 (5)	C13—N5—H4	133 (4)
C4—C2—C1	133.0 (5)	C12—N5—H4	118 (4)
N1—C2—C1	117.3 (5)	C13—N6—C14	106.5 (5)
N1—C3—N2	111.4 (5)	C13—N6—Co2	138.4 (4)
N1—C3—H3	124.3	C14—N6—Co2	113.9 (3)
N2—C3—H3	124.3	Co2—O1W—H1W	111 (5)
C2—C4—N2	105.7 (5)	Co2—O1W—H2W	114 (5)
C2—C4—C5	133.4 (5)	H1W—O1W—H2W	107 (3)
N2—C4—C5	120.3 (5)	Lu1—O2W—H3W	119 (4)
O3—C5—O4	124.3 (5)	Lu1—O2W—H4W	133 (4)
O3—C5—C4	119.3 (5)	H3W—O2W—H4W	108 (3)
O4—C5—C4	116.4 (5)	H5W—O3W—H6W	107 (3)
O6—C6—O5	122.4 (5)		

Symmetry codes: (i) *x*+1, *y*+1, *z*; (ii) −*x*+1, −*y*+1, −*z*+1; (iii) *x*, *y*−1, *z*; (iv) −*x*+1, −*y*+2, −*z*; (v) *x*, *y*+1, *z*; (vi) *x*−1, *y*−1, *z*.

### Hydrogen-bond geometry (Å, °)

**Table:**

*D*—H···*A*	*D*—H	H···*A*	*D*···*A*	*D*—H···*A*
N2—H1···O5^vii^	0.86 (4)	2.04 (4)	2.900 (6)	175 (5)
N2—H1···O6^vii^	0.86 (4)	2.59 (5)	3.139 (6)	122 (4)
O1W—H1W···O8^v^	0.80 (5)	2.04 (5)	2.806 (6)	161 (6)
N4—H2···O1W^iii^	0.85 (4)	2.12 (3)	2.948 (6)	162 (5)
N4—H2···O10	0.85 (4)	2.57 (6)	3.016 (7)	114 (4)
O1W—H2W···O8^viii^	0.81 (5)	1.94 (6)	2.748 (6)	174 (6)
O2W—H3W···O3W	0.81 (5)	1.89 (5)	2.693 (6)	172 (7)
N5—H4···O8^ix^	0.85 (4)	2.23 (4)	3.052 (6)	161 (5)
O2W—H4W···O10^x^	0.80 (6)	2.06 (6)	2.851 (7)	171 (6)
O3W—H5W···O11^iii^	0.86 (5)	2.06 (6)	2.902 (6)	166 (5)
O3W—H6W···O4	0.86 (6)	2.10 (6)	2.928 (6)	163 (6)
C8—H8···O3	0.93	2.44	3.217 (7)	141.
C8—H8···O9	0.93	2.39	3.190 (7)	144.
C13—H13···O2W^xi^	0.93	2.42	3.352 (7)	178.

Symmetry codes: (vii) −*x*+1, −*y*, −*z*+1; (v) *x*, *y*+1, *z*; (iii) *x*, *y*−1, *z*; (viii) −*x*, −*y*+1, −*z*; (ix) −*x*, −*y*, −*z*; (x) *x*+1, *y*, *z*; (xi) −*x*+1, −*y*+1, −*z*.
